# Analogical Reasoning in Children With Autism Spectrum Disorder: Evidence From an Eye-Tracking Approach

**DOI:** 10.3389/fpsyg.2018.00847

**Published:** 2018-05-30

**Authors:** Enda Tan, Xueyuan Wu, Tracy Nishida, Dan Huang, Zhe Chen, Li Yi

**Affiliations:** ^1^Department of Psychology, University of British Columbia, Vancouver, BC, Canada; ^2^Guangzhou Cana School, Guangzhou, China; ^3^Guangzhou Rehabilitation and Research Center for Children with ASD, Guangzhou, China; ^4^Department of Psychology, Arizona State University, Tempe, AZ, United States; ^5^Department of Human Ecology, University of California, Davis, Davis, CA, United States; ^6^Department of Psychology, Beijing Key Laboratory of Behavior and Mental Health, Peking University, Beijing, China

**Keywords:** autism spectrum disorder, analogical reasoning, eye-tracking, executive function, cognition

## Abstract

The present study examined analogical reasoning in children with autism spectrum disorder (ASD) and its relationship with cognitive and executive functioning and processing strategies. Our findings showed that although children with ASD were less competent in solving analogical problems than typically developing children, this inferior performance was attributable to general cognitive impairments. Eye-movement analyses revealed that children with ASD paid less attention to relational items and showed fewer gaze shifts between relational locations. Nevertheless, these eye-movement patterns did not predict autistic children’s behavioral performance. Together, our findings suggest that ASD *per se* does not entail impairments in analogical reasoning. The inferior performance of autistic children on analogical reasoning tasks is attributable to deficits in general cognitive and executive functioning.

## Introduction

Analogical reasoning involves identifying similarities between exemplars and transferring attributes from one exemplar to another exemplar (Gentner, 1983, 2003). This process allows individuals to extract information from the environment and apply the information to new contexts (Holyoak et al., 1984), and serves as a building block for higher-order functions such as categorization (Namy and Gentner, 2002), problem solving (Tunteler and Resing, 2002), metaphor (Gentner, 1988), symbolic understanding (DeLoache, 1987), and social function (Liu et al., 1997; Landau et al., 2010). The present study explored the developmental trajectory of analogical reasoning in children with autism spectrum disorder (ASD).

When identifying correspondences between items, individuals can focus on either shared surface features or shared relational structures. For example, a black dog chasing a ball can be matched with another black dog eating food for their shared physical feature (i.e., black color), or with a bear chasing a chicken for their shared relational structure (e.g., chasing an object) (Richland et al., 2006). Developmental research shows that young children tend to focus on surface features and match items based on physical similarities. In preschool, children undergo a ‘perceptual-to-relational shift’, whereby they pay more attention to underlying attributes and match items based on shared relational structures (Holyoak et al., 1984; Gentner and Toupin, 1986; Gentner, 1988; Rattermann and Gentner, 1998).

What explains this ‘perceptual-to-relational shift’? Prior research has linked this transition to the development of executive functioning skills such as working memory and inhibitory control (Halford, 1993; Waltz et al., 2000; Richland et al., 2006). Specifically, working memory enables individuals to maintain mental representations of items, and inhibitory control allows individuals to suppress perceptual distraction (Richland et al., 2006; Krawczyk et al., 2010). Consistent with these ideas, empirical evidence shows that reduced working memory leads to impaired analogical reasoning: people whose mental resources were depleted (by a working-memory load or by stress) were more likely to map scenes based on perceptual as opposed to relational similarities (Tohill and Holyoak, 2000; Waltz et al., 2000). In a similar vein, individuals whose executive functioning was compromised by frontal brain lobe damage showed a greater tendency to match objects based on perceptual as opposed to relational features (Morrison et al., 2004; Krawczyk et al., 2008). Taken together, these findings demonstrate the central roles of executive functioning in analogical reasoning.

It remains unclear how children with ASD solve analogical problems and whether they tend to match items based on physical or relational similarities. ASD is a neurodevelopmental disorder characterized by impairments in social communication and social interaction, as well as restricted patterns of behavior, interests, or activities (American Psychiatric Association, 2013). There are several, non-mutually exclusive reasons to believe that children with ASD may be less competent in solving analogical problems. First, a converging body of evidence shows that children with ASD have impairments in working memory (Bennetto et al., 1996; Corbett et al., 2009) and inhibitory control (Mosconi et al., 2009; Robinson et al., 2009; for review, see Hill, 2004). Given the crucial roles of working memory and inhibitory control in relational reasoning (Richland et al., 2006; Krawczyk et al., 2010), it is possible that children with ASD may show less mature analogical reasoning patterns and match objects based on perceptual as opposed to relational similarities.

Impaired analogical reasoning in children with ASD is also predicted by the weak central coherence (WCC) theory (Frith and Happé, 1994; Happé and Frith, 2006). This theory posits that individuals with ASD tend to engage in localized, detail-oriented processing and have difficulties extracting global information from the environment. Consistent with this view, past research demonstrates that individuals with ASD pay greater attention to local features. For example, individuals with ASD and Asperger syndrome respond faster than normal controls on the Embedded Figures Test (which assesses participants’ ability to disembed details from context; Jolliffe and Baron-Cohen, 1997; Shah and Frith, 1983), and children with ASD are less capable of applying knowledge to new situations (Peterson and Bowler, 2000). In recent years, new research has cast doubt on the validity of the central coherence construct (O’Laughlin and Thagard, 2000; Pellicano et al., 2005). Other studies show that people with ASD are able to engage in global processing when instructed to (Mottron et al., 1999; Caron et al., 2006). These findings suggest that the tendency to engage in detail-focused processing in individual with ASD is better interpreted as a cognitive style rather than a deficit (Happé, 1999; Happé and Frith, 2006). Despite these controversies, it is possible that the detail-focused bias in individuals with ASD (whether it be a cognitive style or a deficit) may direct their attention toward perceptual features and disrupt relational encoding.

Empirical studies on analogical reasoning in ASD have found mixed results. In an earlier study, Reed (1996) reported that children with ASD performed at lower levels on analogical reasoning tests than did IQ-matched typically developing (TD) children and children with intellectual disabilities. In contrast, other studies have found comparable performance between ASD and control groups when IQ scores were matched (Scott and Baron-Cohen, 1996; Sahyoun et al., 2009; Morsanyi and Holyoak, 2010) and when IQ scores were not matched (Green et al., 2014; Krawczyk et al., 2014). These inconsistent findings may be attributable to differences in study paradigms and participant characteristics.

The current study examined the developmental trajectory of analogical reasoning in children with ASD and explored the roles of cognitive functioning and processing strategies. We used a scene analogy test adapted from Honomichl and Chen (2006). The original version of the paradigm was developed by Markman and Gentner (1993) and has been used to study analogy reasoning in children with ASD (Krawczyk et al., 2014). This paradigm requires the use of semantic memory and knowledge; therefore, it is considered to have greater ecological validity than traditional measures that only require abstract reasoning (Krawczyk et al., 2014). Given that the ‘perceptual-to-relational shift’ occurs in TD children at around 6 years, we used a cross-sectional design comparing analogical reasoning in autistic and TD children at age 6 and 8 years.

To explore the roles of cognitive and executive functioning in analogical reasoning, we compared autistic and TD children’s performance on the scene analogy test and statistically controlled for IQ scores. This approach allowed us to determine whether TD and ASD groups differ in analogical reasoning abilities when cognitive abilities (IQ scores) are not matched, and, if so, whether cognitive differences account for group differences. We reasoned that if analogical reasoning in ASD is affected by cognitive impairments, there should be differences in performance between ASD and TD groups when IQ scores are not controlled for, and no differences when IQ scores are controlled for. In addition to IQ, we also measured children’s working memory, inhibitory control, and cognitive flexibility to explore the roles of executive functioning skills in analogical reasoning.

The current study also examined children’s eye-movements during analogical reasoning tasks and explored the roles of attention and processing strategies in the problem-solving process. It is believed that fixations (the maintenance of visual gaze on specific locations) reflect engagement of attention and processing of the stimulus at the location (for review, see Eckstein et al., 2017), and saccades (rapid eye movement between fixations) between task-relevant locations reflect item comparison, feature extraction, and information integration (Demarais and Cohen, 1998; Thibaut and French, 2016; Eckstein et al., 2017). In the current study, we computed children’s fixation times on relational locations and saccade paths between relationally relevant locations. Further details of these measures are provided in the material and methods section. These two eye-movement measures have been widely used in analogical reasoning research using similar paradigms and stimuli (Demarais and Cohen, 1998; Gordon and Moser, 2007; Thibaut et al., 2011; Chen et al., 2016; Thibaut and French, 2016; Eckstein et al., 2017) and in other research areas with children diagnosed with ASD (Yi et al., 2013, 2014). We reasoned that if children with ASD are biased toward local features and perceptual similarities, they should spend less time looking at relational locations and show fewer saccade paths between relational locations.

## Materials and Methods

### Participants

Participants consisted of four groups of children: 6-year-old ASD (*N* = 13), 8-year-old ASD (*N* = 21), 6-year-old TD (*N* = 25), and 8-year-old TD (*N* = 20). Informed consent was obtained from all participants and/or their guardians. Demographic information for each group is summarized in **Table 1**. Children’s IQ was measured through the Combined Raven’s Test (CRT-C2; Wang et al., 1999). TD and ASD children in the same age groups were matched by age [6-year-old, *t*(17.79) = -0.171, *p* = 0.865; 8-year-old, *t*(39) = 0.34, *p* = 0.738]. The 8-year-old ASD group and 6-year-old TD group were matched by IQ (raw scores on CRT-C2: *t*(44) = -0.03, *p* = 0.98). Children in the TD groups were recruited from a primary school in a metropolitan area of southern China. Children in the ASD group were recruited from a special needs school in the same city and had received a diagnosis of ASD based on DSM-IV (American Psychiatric Association, 1994) by a licensed clinician experienced in the assessment of autism. None of the children in the ASD group were classified as high-functioning autism or received a diagnosis of Asperger syndrome, nor did any children in the ASD or TD groups receive a diagnosis of attention-deficit/hyperactivity disorder (ADHD).

**Table 1:** Means and standard deviations of behavioral performance and eye-movement indices in the scene analogy tasks and executive functioning tasks.

	Index	TD		ASD	
			
		6-year-old	8-year-old	6-year-old	8-year-old
Male/Female		24/1	17/3	12/1	18/3
Age		6.02 (0.80)	8.80 (0.72)	6.08 (1.19)	8.72 (0.75)
Autism spectrum quotient	AQ-child	63.72 (13.08)	60.85 (23.08)	86.54 (14.34)	83.38 (15.05)
Intelligence	Standardized Raven score	95.03 (9.99)	91.43 (12.76)	84.65 (11.85)	69.00 (18.65)
	Raw Raven score	21.48 (9.35)	37.00 (9.30)	15.46 (5.90)	21.38 (12.22)
Scene analogy tasks	Behavioral performance	0.62 (0.19)	0.74 (0.22)	0.49 (0.18)	0.58 (0.16)
	Relational interpretation	5055.51 (2371.34)	5642.04 (2989.10)	3956.96 (1184.51)	3527.11 (1364.54)
	Relational encoding	26.08 (13.96)	34.35 (20.05)	14.00 (7.08)	18.95 (10.80)
Digit recall task	Digit span	5.40 (1.08)	6.55 (1.19)	4.92 (1.32)	4.67 (1.65)
Block recall task	Block span	3.60 (1.00)	4.75 (1.12)	3.00 (1.08)	2.90 (1.09)
Stroop-like tasks	Reaction time (ms)	2745.11 (617.94)	2494.88 (628.02)	4629.42 (1643.72)	2652.59 (1012.54)
DCCS	Rank	2.04 (0.84)	2.65 (0.49)	1.46 (1.13)	1.62 (0.97)
FIST	Shift percentage	0.73 (0.25)	0.83 (0.18)	0.34 (0.31)	0.50 (0.33)


**Standard deviations are shown in parentheses. TD: typically developing; ASD: autism spectrum disorder; DCCS: dimensional change card sort; FIST: Flexible Item Selection Task*.*

To confirm the ASD diagnoses, we assessed the levels of autistic traits using the Chinese version of the Autism Spectrum Quotient (AQ-Child; Auyeung et al., 2008). Children in both ASD groups received scores above the cut-off score of 76 (*M*_6-year-olds_ = 86.54; *M*_8-year-old_ = 83.38), and children in both TD groups received scores below the cut-off score of 76 (M_6-year-olds_ = 63.72; *M*_8-year-old_ = 60.85). A 2 (group: ASD vs. TD) × 2 (age: 6-year -old vs. 8-year-old) ANOVA on AQ revealed that participants in the ASD group received significantly higher AQ scores than participants in the TD group, *F*(1,75) = 33.87, *p < 0*.001, η^2^ = 0.31. No effects of age or the interaction between group and age were found.

### Procedures and Materials

We measured children’s analogical reasoning skills using a scene analogy task developed by Honomichl and Chen (2006), and recorded children’s eye-movements during the task using a Tobii X60 eye-tracker (sampling rate: 60 Hz). Children’s executive function skills were measured in a different session using the digit recall task (Gathercole et al., 2004), the block recall task (Corsi, 1972; Kessels et al., 2000), the Day–Night and Happy-Sad Stroop tasks (Gerstadt et al., 1994; Lagattuta et al., 2011), the dimensional change card sort task (DCCS; Zelazo, 2006), and the Flexible Item Selection Task (FIST; Jacques and Zelazo, 2001). All procedures were in accordance with the ethical standards of the institutional research committee and with the 1964 Helsinki declaration and its later amendments or comparable ethical standards.

#### Scene Analogy Task

**FIGURE 1:**
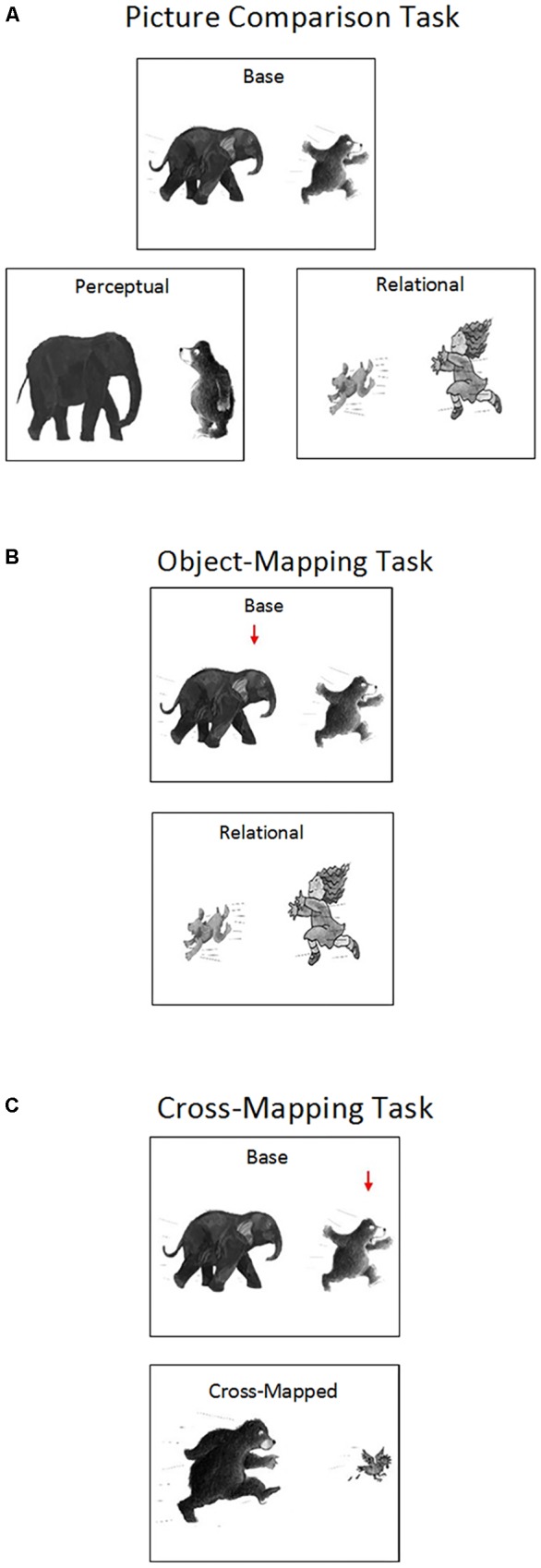
Examples of Areas of Interest (AOIs) and saccade paths in the Picture Comparison Task **(A)**, the Object-Mapping Task **(B)**, and the Cross-Mapping Task **(C)**.

The scene analogy task involved six sets of pictures depicting different relations, including *chasing, feeding, pulling, riding, throwing*, and *washing*. The relations between objects were clearly depicted in each picture (see **Figure 1**). Each set included four types of pictures: (1) a base picture showing one object (object A) acting on another object (object B) (e.g., an elephant chasing a bear), (2) a perceptually similar picture showing the same objects (object A and object B) but no interactions (e.g., an elephant standing beside a bear), (3) a relationally similar picture showing the same relation as the base picture with two new objects (object C and object D) (e.g., a child chasing a dog), and (4) a cross-mapped picture showing the same relation as the base picture with a base picture object (object B) and a new object (object E). Crucially, the base picture object (object B) played the opposite role (e.g., a bear chasing a chicken) in the cross-mapped picture. The pictures were presented on a 17″ (34.3 cm × 25.8 cm) laptop screen (HP Elitebook) at a 1024 × 768 pixel resolution. The size of each image was 12.7 cm × 10.1 cm (11 × 9° of visual angle at a testing distance of 65 cm).

#### Practice Trials

Children first completed 8 practice trials and learned to use a green paper arrow to indicate their choice. As the focus of the study was on autistic and TD children’s spontaneous matching strategies (as opposed to their ability to learn from experimenter the “right way” of mapping pictures/objects), we provided no feedback to children’s responses in both practice trials and test trials. The first 4 trials of the practice section involved three pictures: a base picture at the top, and a perceptually similar picture and a relationally similar picture at the bottom. The experimenter placed a red paper arrow on the base picture, gave the child a green paper arrow, and said, “Can you place this green paper arrow on one of the bottom pictures so that it goes with the picture on the top (pointing toward the red arrow and the top picture)? Which one of these two pictures goes with the top one? Why does this picture go with the top one?” The experimenter repeated the instructions if children showed signs of confusion. No other feedback was provided.

The last four trials of the practice section involved two pictures: a base picture at the top and a relationally similar picture or a cross-mapped picture at the bottom (see object-mapping and cross-mapping tasks in **Figure 1**). The base picture showed a “virtual” red arrow pointing toward one of the objects, designating the target object of the trial. For base pictures presented with relationally similar pictures, the target was randomly chosen between the two objects (i.e., Object A or B). For base pictures presented with cross-mapped pictures, the arrow was always placed on the object that played a different role in the cross-mapped picture (i.e., object B). The experimenter gave the child a green paper arrow, and said, “Look, there is a red arrow (pointing to the “virtual” red arrow on the screen) in this picture. Can you place this green paper arrow on one of these two objects here (pointing to the two objects in the relationally similar picture or the cross-mapped picture) so that it goes with the target object here (pointing back to the target/red arrow in the base picture)? Which object goes with this one? Why does the object go with this one?” Children were asked to first view the pictures for 8 s and refrain from responding. Eight seconds later, children were allowed to provide answers. The experimenter corrected the children if they placed the green paper arrow outside of the pictures or if they responded within the 8-s viewing period. No other feedback was given.

#### Test Trials

Test trials began after the practice trials. The eye-tracker was calibrated using a five-point calibration program (Tobii Technology AB, Danderyd, Sweden). A wooden stick was used to direct children’s attention to the computer screen. Calibrations were considered successful when all five points showed good fit in the computed mapping for both eyes (error vectors <0.5° of visual angle). After calibration, children completed six blocks of test trials, each block consisting of a picture comparison task, an object-mapping task, and a cross-mapping task. Participants were randomly assigned to two testing orders. Half of them viewed pictures in the following order: *chasing, feeding, pulling, riding, throwing*, and *washing*; the other half viewed pictures in the reverse order. The length of each task was about 5 min and the total length of the scene analogy task was about 15 min. Children’s behavioral and verbal responses were manually recorded and videotaped. The experimenter provided no feedback to children’s responses.

In the picture comparison task, children first viewed the base, perceptually similar, and relationally similar pictures separately and were asked to describe the content of each picture (“What’s happening in this picture?”). Afterwards, all three pictures were presented on the screen, with the base picture at the top and the perceptually similar picture and the relationally similar picture at the bottom (see **Figure 1**). Children were asked to first view the pictures for 8 s and refrain from responding while their eye-movements were recorded. Afterwards, the experimenter asked children to place a green paper arrow on one of the two bottom pictures so that the selected picture went with the base picture (“Can you place this green paper arrow on one of the bottom pictures so that it goes with the picture on the top? Why does this picture go with the picture on the top?”). The experimenter provided no feedback to children’s responses.

In the object-mapping task, children first viewed the base picture and the relationally similar picture separately and described the content of each picture. Afterwards, the two pictures were shown together on the screen, with the base picture at the top and the relationally similar picture at the bottom. In the base picture, a “virtual” red arrow was placed on one of the two objects, designating the target object (see **Figure 1**). The placement of the arrow (i.e., on Object A or B) was randomized across trials and participants. Children were asked to view the pictures for 8 s and refrain from responding while their eye-movements were recorded. Eight seconds later, children were asked to place a green paper arrow on one of the objects in the relationally similar picture so that the selected object went with the target object (“Can you place this green paper arrow on one of these two objects so that it goes with the object with a red arrow? Why does the object go with this one?”). The experimenter provided no feedback to children’s responses.

In the cross-mapping task, children first viewed the base picture and the cross-mapped picture separately and described the content of each picture. Then the two pictures were shown together on the screen, with the base picture at the top and the cross-mapped picture at the bottom. A “virtual” red arrow was placed on one of the objects in the base picture, designating the target object (see **Figure 1**). Crucially, the target object also appeared in the cross-mapped picture but played the opposite role (e.g., being chased versus chasing). Children were asked to view the pictures for 8 s and refrain from responding while their eye-movements were recorded. Afterwards, children were asked to place a green paper arrow on one of the objects in the cross-mapped picture so that the selected object went with the target object (“Can you place this green paper arrow on one of these two objects so that it goes with the object with a red arrow? Why does the object go with this one?”). The experimenter provided no feedback to children’s responses.

#### Eye-Tracking Measures

To explore mental processes underlying analogical reasoning, we examined children’s eye-movements during scene analogy tasks. Three areas of interest (AOIs) were defined for each task. For the picture comparison task, we defined one AOI covering the base picture, one covering the perceptually similar picture, and one covering the relationally similar picture. For the object-mapping and cross-mapping tasks, we defined one AOI covering the target object, one covering the correct object, and one covering the incorrect object (see **Figure 1**). Relational locations were defined as AOIs covering relational pictures and objects, including the relationally similar picture in the picture comparison task and the correct objects in the object-mapping and cross-mapping tasks. Non-relational locations were defined as AOIs covering non-relational pictures and objects, including the perceptually similar picture in the picture comparison task and the incorrect objects in the object-mapping and cross-mapping tasks.

To examine attention distribution, we computed children’s fixation times on relational and non-relational locations. Fixation times were extracted using Tobii Studio’s default fixation filters. The average proportion of missing data (e.g., eye blinks and gaze time off the screen) was 17.51%. The amount of missing data did not differ by group, *F*(1,75) = 4.97, *p* = 0.029, η^2^ = 0.062, or age, *F*(1,75) = 3.24, *p* = 0.076, η^2^ = 0.041, nor was there a Group × age interaction, *F*(1,75) = 0.49, *p* = 0.488, η^2^ = 0.006. We reasoned that fixation time spent on relational locations represented relational interpretation and processing of relational stimuli (Eckstein et al., 2017).

Saccade paths (fixation sequences) were defined as gaze shifts between fixations on AOIs. To examine processing strategies, we computed the frequency of saccade paths between target and relational locations and saccade paths between target and non-relational locations using a custom-made Scanpath MATLAB toolbox. We reasoned that saccade paths between target and relational locations represented item comparison and relational encoding (Chen et al., 2016). These fixation-based and saccade-based measures have been widely used in analogical reasoning research using similar paradigms and stimuli (Demarais and Cohen, 1998; Gordon and Moser, 2007; Thibaut et al., 2011; Chen et al., 2016; Thibaut and French, 2016), and in other research areas with children diagnosed with ASD (Yi et al., 2013, 2014).

#### Digit Recall Task and Block Recall Task

Working memory was measured using the digit recall task and the block recall task. In the digit recall task, children verbally recalled sequences of random digits. The digits were orally presented by an experimenter at the rate of one digit per second (Gathercole et al., 2004). Four sequences of digits were presented at each length. The length increased by one digit after the child passed the previous length by recalling at least two out of four sequences. The test ended if the child incorrectly recalled three out of the four sequences at a given length. Digit span was defined as the longest length the child had achieved.

The block recall task (Corsi, 1972; Kessels et al., 2000) involved nine wooden cubes (25 mm × 25 mm × 25 mm) placed on a board (30 cm × 21 cm) in a scattered array. The experimenter tapped a sequence of blocks at the rate of one block per second and then asked the child to reproduce the sequence in the same order. The task started with a two-block sequence. The length of the sequence increased by one if the child passed the previous length. The test ended if child was unable to reproduce two out of three trials at a given length. Block span was defined as the longest length the child had achieved.

#### Stroop-Like Tasks

Children’s inhibitory control was measured using two Stroop-like tasks (Gerstadt et al., 1994; Lagattuta et al., 2011). The day–night task involved a set of cards with cartoon pictures depicting either a sun or a moon. The cards were presented in a random order on a computer screen. Children were asked to say “day” when they saw the moon and “night” when they saw the sun. The task started with a four-trial practice block. The test block began if the child passed three out of four practice trials. The test block consisted of 16 trials. The happy-sad task followed the same procedure as the day–night task, except that the sun and the moon were replaced by a happy face and a sad face. Children were asked to say “happy” when they saw the sad face and “sad” when they saw the happy face. Children’s reactions in these two tasks were videotaped and coded by two trained research assistants (interrater reliability *r* = 1.00). A response was coded as correct only if the correct answer was given in the first place; self-corrected answers were coded as incorrect.

#### DCCS Task

We used the DCCS task (Zelazo, 2006) to measure children’s cognitive flexibility. The task consisted of a pre-switch phase, a post-switch phase, and a border phase. Children were presented with a set of cards with pictures that varied in two dimensions (color and shape). Children were asked to sort the cards based on one dimension (e.g., color) in the pre-switch phase, and then based on a different dimension (e.g., shape) in the post-switch phase. The border phase began after children passed the pre-switch phase and the post-switch phase by correctly sorting at least 5 out of 6 sets of cards in each phase. In the border phase, the sorting rule depended on whether there was a border in the picture: if there was a border, the cards should be sorted by color; if there was no border, the cards should be sorted by shape. To pass the border phase, children needed to correctly sort at least 9 out of 12 sets of cards. DCCS rank was defined as the number of phases children passed: if they failed the pre-switch phase, the DCCS rank was scored as 0; if they only passed the pre-switch phase, the DCCS rank was scored as 1; if they passed the pre- and post-switch phase, the DCCS rank was scored as 2; if they passed all phases, the DCCS rank was scored as 3.

#### FIST

Children’s cognitive flexibility was measured using the FIST (Jacques and Zelazo, 2001). The task involved 15 sets of cartoon cards. Each set included three cards with each card representing a combination of four dimensions (size, shape, color, and number). In each set, two cards matched each other on one dimension (e.g., color) while another pair of cards matched each other on a different dimension (e.g., shape). The task consisted of a demonstration phase and a test phase. In the demonstration phase, the experimenter picked out two pairs of cards and demonstrated how the cards in each pair matched each other. In the test phase, children were asked to pick out two cards that matched each other on one dimension. Then the experimenter shuffled the cards and asked children to pick a new pair of cards that matched each other on a different dimension. Shift percentage was defined as the percentage of trials in which children correctly matched two pairs of cards (Yerys et al., 2012).

## Results

### Analysis Plan

Data analyses were conducted using IBM SPSS Version 21 (SPSS Inc, Chicago, IL, United States) to address the following questions: (1) group and age differences in cognitive and executive functioning, (2) group and age differences in behavioral performance on scene analogy tasks, (3) group and age differences in eye-movement patterns (reflecting processing strategies), and (4) the relationships between cognitive skills, processing strategies, and behavioral performance. Given that the ASD and TD groups in the same age groups were not matched by intelligence, we conducted two additional analyses to examine group differences while controlling for IQ: First, we performed a series of ANCOVAs controlling for children’s standardized IQ scores. The effects found in these analyses reflected differences that were not attributable to intelligence. Second, we performed two-sample *t*-tests between 6-year-old TD and 8-year-old ASD groups; children in these groups were matched by IQ (but not age). These analyses provide further information about whether differences between the ASD and TD groups were attributable to intelligence. When the assumption of normality was violated, we transformed raw scores into ranks and performed supplementary tests on rank data.

### Cognitive Skills and Executive Functioning

**Table 1** provides descriptive statistics of children’s performance on cognitive and executive functioning tasks. To examine group and age differences in cognitive skills, we performed 2 (group: ASD vs. TD) × 2 (age: 6-year-old vs. 8-year-old) ANOVA on IQ scores. Shapiro–Wilk test of normality revealed that standardized and raw IQ scores were normally distributed, *p* > 0.05. A 2 (group: ASD vs. TD) × 2 (age: 6-year-old vs. 8-year-old) ANOVA on standardized scores on CRT-C2 revealed main effects of group, *F*(1,69) = 22.78, *p* > 0.001, η^2^ = 0.25, and age, *F*(1,69) = 7.84, *p* = 0.007, η^2^ = 0.102, but no interaction between group and age. An ANOVA on raw scores on CRT-C2 revealed similar group and age differences, *p*s < 0.05. These results revealed that children in the TD and older age groups had higher cognitive skills than children in the ASD and younger age groups, respectively. ANOVA analyses on executive functioning scores revealed that children in the TD group outperformed children in the ASD group on digit recall span, *F*(1,75) = 14.92, *p* < 0.001, η^2^ = 0.166, block recall span, *F*(1,75) = 24.43, *p* < 0.001, η^2^ = 0.246, Stroop reaction time, *F*(1,75) = 20.95, *p* < 0.001, η^2^ = 0.218, DCCS rank, *F*(1,75) = 16.26, *p* < 0.001, η^2^ = 0.178, and FIST shift percentage, *F*(1,75) = 33.65, *p* < 0.001, η^2^ = 0.310. Correlational analyses revealed that standardized IQ scores were positively correlated with digit recall span, *r*(72) = 0.50, *p* < 0.001, block recall span, *r*(72) = 0.50, *p* < 0.001, DCCS rank, *r*(72) = 0.40, *p* < 0.001, and FIST shift percentage *r*(72) = 0.43, *p* < 0.001.

### Behavioral Performance on Scene Analogy Tasks

**FIGURE 2:**
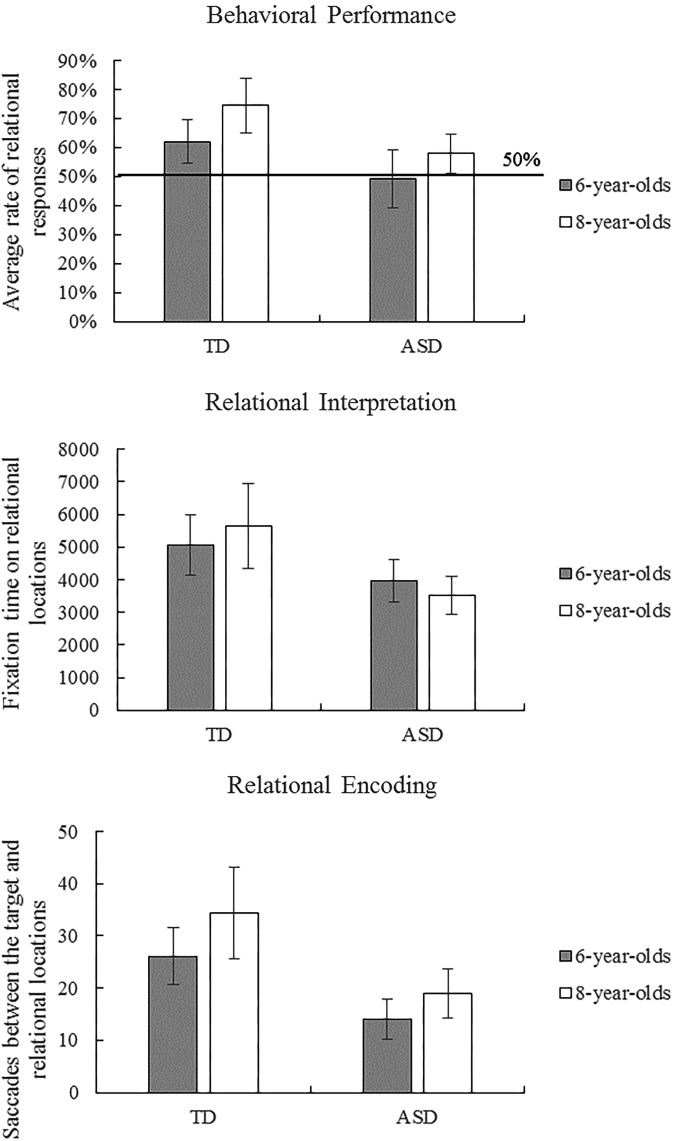
Autism spectrum disorder (ASD) and typically developing (TD) children’s average behavioral performance and eye-movement patterns across three scene analogy tasks. Error bars represent 95% CI of the mean.

Children’s behavioral responses were coded as relational (correct) or non-relational (incorrect). **Table 1** provides descriptive statistics of behavioral performance and eye-movement indices in the scene analogy tasks. **Figure 2** illustrates ASD and TD children’s average behavioral performance across three scene analogy tasks. Average rates of relational responses across tests were above chance (50%) for the 6-year-old TD group, *t*(24) = 3.08, *p* = 0.005, η^2^ = 0.28, 8-year-old TD group *t*(19) = 5.07, *p* < 0.001, η^2^ = 0.57, and the 8-year-old ASD group, *t*(20) = 2.32, *p* = 0.031, η^2^ = 0.20. The 6-year-old ASD group did not perform above chance, *t*(12) = -0.17, *p* = 0.868, η^2^ = 0.00. The Shapiro–Wilk test of normality revealed that children’s behavioral performance deviated significantly from a normal distribution, *p* = 0.005. To better illustrate the nature of the data, we performed ANOVAs, ANCOVAs, and *t*-tests on both raw scores and ranks.

To examine group and age differences, we performed a 2 (group: ASD vs. TD) × 2 (age: 6-year-old vs. 8-year-old) ANOVA on behavioral performance on analogy tasks. The ANOVA revealed main effects of group, *F*(1,75) = 11.24, *p* = 0.001, η^2^ = 0.13, and age, *F*(1,75) = 5.88, *p* = 0.018, η^2^ = 0.073, but no interaction between group and age. A supplementary ANOVA on ranks revealed similar results: main effects of group, *F*(1,75) = 8.61, *p* = 0.004, η^2^ = 0.10, and age, *F*(1,75) = 5.35, *p* = 0.023, η^2^ = 0.067, but no interaction between group and age. These results suggest that, without controlling for intelligence, TD and older children were more likely than autistic and younger children, respectively, to match pictures and objects based on relational as opposed to perceptual similarities.

Given that children in the ASD and TD groups were not matched by intelligence, it is possible that the previously observed group differences were due to general intelligence differences as opposed to ASD-specific characteristics. To control for individual differences in cognitive abilities, we conducted a 2 (group: ASD vs. TD) × 2 (age: 6-year-old vs. 8-year-old) ANCOVA with standardized scores on the Raven test as a covariate. The results showed significant effects of age, *F*(1,68) = 6.52, *p* = 0.013, η^2^ = 0.088, and intelligence, *F*(1,68) = 4.18, *p* = 0.045, η^2^ = 0.058. Crucially, the effect of group did not reach significance, *F*(1,68) = 3.73, *p* = 0.058, η^2^ = 0.052, nor did the interaction between group and age. A supplementary ANCOVA on ranks showed similar results: main effects of age, *F*(1,68) = 5.92, *p* = 0.018, η^2^ = 0.080, and intelligence, *F*(1,68) = 4.33, *p* = 0.041, η^2^ = 0.060, but no effects of group and the interaction between group and age. Consistent with these findings, two-sample *t*-tests between 6-year-old TD and 8-year-old ASD children (who were IQ-matched) showed no significant differences in behavioral performance in raw scores, *t*(44) = 0.77, *p* = 0.446, or ranks, *t*(44) = 0.51, *p* = 0.616. These results suggest that the differences between ASD and TD groups in behavioral performance on analogical reasoning tasks were due to differences in cognitive abilities as opposed to non-cognitive characteristics that are specifically related to ASD.

### Processing Strategies and Eye-Movement Analyses

Although we found no differences in behavioral performance between the ASD and TD groups when IQ was controlled for, it is possible that autistic and TD children process analogical questions in different ways. To explore differences in processing strategies between the ASD and TD groups, we performed ANCOVAs on eye-movement measures (fixation times and saccade paths) with group (ASD vs. TD) and age (6-year-old vs. 8-year-old) as independent variables and with standardized scores on the Raven test as a covariate. **Figure 2** illustrates ASD and TD children’s eye-movement results across three scene analogy tasks.

### Fixation Times

The Shapiro–Wilk tests of normality revealed that fixation times on relational locations and non-relational locations were normally distributed, *p*s > 0.05. The ANCOVA with total fixation time on relational locations (**Figure 2**) revealed a main effect of group, *F*(1,68) = 6.364, *p* = 0.014, η^2^ = 0.086, but no main effects of age, intelligence nor interaction between age and group. In contrast, the ANCOVA with total fixation time on non-relational locations revealed no main effects or interactions, *p*s > 0.05. Consistent with these findings, two-sample *t*-tests showed that 6-year-old TD looked longer than 8-year-old ASD children at relational locations, *t*(39.32) = 2.73, *p* = 0.009, but not at non-relational locations, *t*(44) = 1.83, *p* = 0.074. These results indicate that TD children fixated longer on relational locations (but not on non-relational locations) than did children with ASD, suggesting that they paid more attention to relational items and spent longer time interpreting the relational meaning of the items.

### Saccade Paths

The Shapiro–Wilk tests of normality revealed that frequencies of saccades between the target and non-relational locations were normally distributed, *p* > 0.05, whereas frequencies of saccades between the target and relational locations deviated significantly from a normal distribution, *p* = 0.006. The ANCOVA with saccades between the target and the relational location (**Figure 2**) revealed a main effect of group, *F*(1,68) = 8.022, *p* = 0.006, η^2^ = 0.106, but no main effects of age (*p* = 0.054), intelligence, or interaction between age and group. A supplementary ANCOVA on ranks showed similar results: significant effects of group, *F*(1,68) = 5.79, *p* = 0.019, η^2^ = 0.078, but no effects of age, intelligence, or the interaction between age and group. The ANCOVA with saccades between the target and the non-relational location revealed no main effects or interactions, *p*s > 0.05. In a similar vein, two-sample *t*-tests between 6-year-old TD and 8-year-old ASD children showed a marginally significant effect for saccades between the target and the relational location, *t*(43.74) = 1.95, *p* = 0.058, but no effects for saccades between the target and the non-relational location, *t*(44) = 0.82, *p* = 0.419. These results demonstrate that TD children engaged in more gaze shifts between the target and the relational location than did children in the ASD group, suggesting that TD children spent more time analyzing and encoding the correspondence between pictures/objects than did children with ASD.

Taken together, fixation and saccade analyses suggest that although autistic and TD children performed similarly on scene analogy tasks when intelligence was controlled for, they processed the problems in different ways. Compared with TD children, children with ASD paid less attention to relational locations and showed fewer eye-movement patterns representing relational encoding and mapping. These eye-movement characteristics were not attributable to general cognitive factors and hence may be specifically related to the symptoms of ASD.

### Regressions

To explore the relationships between cognitive skills, processing strategies, and behavioral performance, we conducted three-step hierarchical regressions with behavioral performance on scene analogy tasks as the dependent variable. Given that children in the TD and ASD groups showed different processing strategies but achieved similar behavioral performance, it is possible that the relationships between cognitive and executive functions, processing strategies, and behavioral performance may show different patterns between groups. Therefore, we conducted separate regression analyses for the TD and ASD groups. In Model 1, we entered age and AQ to control for demographic differences within ASD and TD groups. In Model 2, we entered cognitive and executive functioning measures including standardized Raven score, digit recall span, block recall span, Stroop reaction time, DCCS rank, and FIST shift percentage. Model 3 contained eye-movement measures representing processing strategies, including relational interpretation (fixation time on relational locations) and relational encoding (saccade paths between the target and the relational location).

Results are displayed in **Table 2**. For the TD group, age and AQ accounted for 20% of the variation in behavioral performance, *F*(2,39) = 4.95, *p* = 0.012. Adding cognitive measures to the model explained an additional 20% of the variation, *F*(6,33) = 1.78, *p* = 0.134. Finally, the addition of eye-movement measures accounted for an additional 19% of the variation, *F*(2,31) = 7.18, *p* = 0.003. Importantly, after statistical control of all covariates, higher relational interpretation (fixation time on relational locations) predicted higher behavioral performance for the TD group, *β* = 0.52, 95% CI [0.13, 0.90], *t*(31) = 2.72, *p* = 0.011. For the ASD group, age and AQ accounted for 1% of the variation in behavioral performance, *F*(2,28) = 0.14, *p* = 0.874. Adding cognitive measures to the model explained an additional 41% of the variation, *F*(6,22) = 2.61, *p* = 0.046. Finally, the addition of eye-movement measures accounted for an additional 1% of the variation, *F*(2,20) = 0.08, *p* = 0.924. After statistical control of all covariates, higher IQ predicted higher behavioral performance for the ASD group, *β* = 0.74, 95% CI [0.11, 1.37], *t*(20) = 2.46, *p* = 0.023. These results suggest that behavioral performance on scene analogy tasks was determined by different factors between the ASD and TD groups: for TD children, relational interpretation explained most of the individual differences, whereas for children with ASD, intelligence was the most robust predictor of behavioral performance.

**Table 2:** Regressions of behavioral performance on scene analogy tasks.

	TD			ASD		
		
	**β* (Step 1)*	**β* (Step 2)*	**β* (Step 3)*	**β* (Step 1)*	**β* (Step 2)*	**β* (Step 3)*
Predictor						
Age	0.43^∗∗^	0.08	0.13	0.06	0.23	0.19
AQ	0.15	0.19	0.10	-0.07	0.09	0.08
IQ		-0.02	0.06		0.74^∗^	0.74^∗^
Digit recall span		0.39^∗^	0.22		-0.46	-0.46
Block recall span		-0.05	-0.09		-0.02	-0.01
Stroop reaction time		-0.15	-0.04		-0.22	-0.25
DCCS rank		0.15	0.20		-0.12	-0.11
FIST shift percentage		0.18	0.15		0.36	0.35
Relational interpretation			0.52^∗^			-0.11
Relational encoding			-0.03			0.07
*R*^2^	0.20	0.40	0.59	0.01	0.42	0.43
Δ*R*^2^		0.20	0.19		0.41	0.01
*F* statistic	*F*(2,39) = 4.95,	*F*(6,33) = 1.78,	*F*(2,31) = 7.18,	*F*(2,28) = 0.14,	*F*(6,22) = 2.61,	*F*(2,20) = 0.08,
	*p* = 0.012	*p* = 0.134	*p* = 0.003	*p* = 0.874	*p* = 0.046	*p* = 0.924


**TD: typically developing; ASD: autism spectrum disorder; DCCS: dimensional change card sort; FIST: Flexible Item Selection Task*; *β*: standardized regression coefficient; *R*^*2*^: coefficient of determination; Δ*R*^*2*^: change in coefficient of determination. ^∗^*p* < 0.05, ^∗∗^*p* < 0.01.*

## Discussion

The present study examined autistic children’s understanding of relational similarities by comparing their performance on a scene analogy task with age-matched TD children. The results showed that, when IQ was not matched between groups, children with ASD were less likely to match items based on relational similarities than were TD children. However, after controlling for IQ, there were no significant differences between the ASD and TD groups. These results suggest that children with ASD were less competent in solving analogical problems than were TD children, and this reduced analogical reasoning ability may be attributable to impairments in general cognitive functioning.

Participants’ behavioral performance showed a developmental trend: older children in both the ASD and TD groups were more likely than younger children to match items based on relational rules, even after controlling for IQ. These results are consistent with age-related changes observed in prior studies with TD children (Waltz et al., 2000; Honomichl and Chen, 2006; Richland et al., 2006) and children with ASD (Morsanyi and Holyoak, 2010; Green et al., 2014), suggesting that the “relational shift” (Rattermann and Gentner, 1998) occurs in both the TD and ASD groups. Indeed, the fact that there were no significant interactions between age and group suggests that children with ASD undergo similar developmental changes as TD children, but this process may be hampered by autistic children’s cognitive impairments and hence shows a lagging trajectory.

Eye-movement data provide further insight into children’s mental processes during analogical reasoning. Although children in the ASD and TD groups were similar in behavioral performance, they showed different attentional patterns and processing strategies. Specifically, TD children spent more time looking at relational items and showed more saccades between the target and relational locations than did children with ASD. In contrast, no group differences were found in looking time at non-relational items or saccades between the target and non-relational locations, suggesting that the differences between ASD and TD groups in attention and processing strategies were specific to relational locations. These results imply that children with ASD spend less time interpreting and encoding relational correspondences. Importantly, the effect of group reached statistical significance even when IQ was controlled for, suggesting that the differences in eye-movement patterns between ASD and TD are not attributable to general cognitive factors. Thus, these atypical eye-movement patterns may be specific to the symptoms of ASD. Furthermore, eye-movement indices did not differ between age groups for either autistic or TD children, suggesting that attentional patterns and processing strategies remain stable between 6 and 8 years of age.

How do cognitive factors and eye-movement patterns relate to behavioral performance? Regression analyses revealed that the relationships between cognitive and executive skills, processing strategies, and behavioral performance differed between the ASD and TD groups. For the TD group, relational interpretation (fixation time on relational locations) was the only significant predictor in the final model after controlling for demographic factors and cognitive and executive functioning. In contrast, for the ASD group, IQ was the only significant predictor in the final model. These results suggest that analogical reasoning is determined by different factors between the ASD and TD groups: For the TD group, processing strategies play an important role, whereas for the ASD group, intelligence is the most significant factor explaining individual differences. These results, together with the patterns observed in ANCOVAs, support the idea that autistic children’s analogical reasoning skill is constrained by cognitive deficits.

Taken together, our findings support the hypothesis that autistic children’s reduced relational reasoning is attributable to impairments in intellectual and executive functioning. This idea is consistent with past research showing the importance of cognitive and executive functioning in relational reasoning (Halford, 1993; Richland et al., 2006). Indeed, it has been shown that deficiencies in working memory (Waltz et al., 2000), executive control (Tohill and Holyoak, 2000), and frontal lobe functioning (Morrison et al., 2004) lead to reduced relational mapping. Our data also suggest that autistic children’s behavioral performance is not influenced by their tendencies to focus on local features. Although children with ASD paid less attention to relational items and displayed less relational encoding, these eye-movement indices did not predict behavioral performance. The lack of links between autistic children’s eye-movement patterns and behavioral performance implies the existence of compensatory mechanisms allowing autistic children to achieve high performance despite their atypical processing strategies. Future studies should explore these potential compensatory mechanisms.

In the current study, children were not explicitly instructed to find relational similarities, nor did they receive feedback on their responses. Thus, children’s reactions in our study represented spontaneous (as opposed to effortful) analogical reasoning (Gick and Holyoak, 1983). Past research has shown that normal people engage in analogical reasoning spontaneously (Read, 1987; Liu et al., 1997). Our results extend these findings and provide evidence that children with ASD are also able to engage in spontaneous analogical reasoning. Future research should examine whether providing feedback to autistic children facilitates their understanding of relational similarities. Given autistic children’ impaired social functioning, it is possible that they are less sensitive to verbal feedback than TD children.

## Conclusion

The current study provides evidence that although autistic children are less competent in solving analogical problems than are TD children, these differences are explained by autistic children’s impaired cognitive abilities and are not related to their atypical attentional patterns or processing strategies. These results suggest that ASD *per se* does not necessarily imply impairments in analogical reasoning. Our findings should be interpreted in light of several limitations. First, our participants only represented a small range of the autism spectrum. It is possible that autistic children on the lower or higher end of the spectrum will show different behavioral performance and eye-movement patterns. Hence, it is an important future step to recruit participants from a wider range of the spectrum and examine whether reasoning abilities and eye-movement patterns vary as a functioning of the severity of ASD. Future studies should also increase sample sizes to better assess the sizes of the effects. Second, the current study used statistical methods (rather than participant matching) to control for group differences in intelligence. Although this approach allowed us to directly assess the impact of cognitive factors, future studies should use participant matching to better assess group differences beyond cognitive factors.

## Ethics Statement

This study was carried out in accordance with the ethics guidelines of Frontiers in Psychology. The parents or legal guardians of the child participants gave written informed consent in accordance with the Declaration of Helsinki. The children also gave oral assent prior to participation. The protocol was approved by the SYSU Ethics Committee.

## Author Contributions

LY, ZC, and ET conceived and planned the experiments. XW and DH assisted with data collection. ET analyzed the data and wrote the manuscript with support from LY, ZC, and TN.

## Conflict of Interest Statement

The authors declare that the research was conducted in the absence of any commercial or financial relationships that could be construed as a potential conflict of interest.
